# Are medicinal plants polluted with phthalates?

**DOI:** 10.1186/2008-2231-21-43

**Published:** 2013-05-29

**Authors:** Soodabeh Saeidnia, Mohammad Abdollahi

**Affiliations:** 1 Medicinal Plants Research Center, Faculty of Pharmacy, Tehran University of Medical Sciences, Tehran 1417614411, Iran; 2 Department of Toxicology and Pharmacology, Faculty of Pharmacy and Pharmaceutical Sciences Research Center, Tehran University of Medical Sciences, Tehran 1417614411, Iran

## Abstract

Phthalic acid esters (PAEs) have been employed in polymer materials as a plasticizer to form them more flexible, adhesive, and soluble. These compounds are mainly used in paints, varnishes, personal cares, cosmetics, paper coatings, and adhesives even in bottled waters, shampoo, body deodorant, hairspray, and gels. Phthalates are able to possess remarkable toxic variations depending on their structures. So far, Di-(2-EthylHexyl) Phthalate DEHP and Di-n- Butyl Phthalate DBP have been found to cause reproductive and developmental toxicities. The U.S. Environmental Protection Agency (EPA) classified DEHP as probable human carcinogen. To the best of our knowledge, phthalates showed diverse toxicity profiles according to their structures in the liver, kidneys, thyroid, and testes, which are involved in general toxicity. Furthermore, they are introduced as hormonally-active agents, because they can interfere with the endocrine system in human. Incidence of developmental abnormalities (like skeletal malformations and cleft palate, and undescended testes, lowering testes weight and anogenital distance) seems increasing via high exposure to phthalate metabolites. Although, increasing the capacity for phthalate free plasticizer productions is the first step to restrict the distribution of these toxic manmade compounds, finding the new ways for phthalate absorption from the soil in agricultural fields may have benefits. Also, evaluation and examination of diverse sources of medicinal and food plants to determine the level of phthalate accumulation in their organs are extremely recommended to avoid creating toxicity particularly in reproductive systems.

## 

Phthalic acid esters (PAEs) have been employed in polymer materials as a plasticizer to form them more flexible, adhesive, and soluble. These compounds are mainly used in paints, varnishes, personal cares, cosmetics, paper coating, and adhesives even in bottled waters, shampoo, body deodorant, hairspray and gels [1]. Di-n-Butyl Phthalate (DBP) and Di-(2-EthylHexyl) Phthalate (DEHP) are two remarkable and mostly applied ones, which can be released into the environment during production and processing through wastewater. It is reported that the EU produced about 10,000 tons of DBP and 341,000 tons of DEHP during 2007. They can contaminate the agricultural soils through the air as well as oil leakages from farm machinery or organic fertilizers. In soils and sediments, DEHP persists and shows high potential for bioaccumulation [2].

Phthalates are able to possess remarkable toxic variations depending on their structures. So far, DEHP and DBP have been found to cause reproductive and developmental toxicities. The U.S. Environmental Protection Agency (EPA) classified DEHP as probable human carcinogen. To the best of our knowledge, phthalates showed diverse toxicity profiles according to their structures in the liver, kidneys, thyroid, and testes, which are involved in general toxicity. Furthermore, they are introduced as hormonally-active agents, because they can interfere with the endocrine system in human [3].

Incidence of developmental abnormalities (like skeletal malformations and cleft palate, and undescended testes, lowering testes weight and anogenital distance) seems increasing *via* high exposure to phthalate metabolites [3]. The important concern around phthalates, anti-androgenic effect is associated with human reproductive system, such as affecting sperm counts and histopathological alterations in the testes leading to male infertility. In addition, literature review reveals that there is a correlation between phthalate metabolite concentrations in maternal breast milk and sex hormone concentration in male offspring. Furthermore, fetal exposure to phthalate shows a relation with behavior and mental ability (Figure 1). For instance, in a study on pregnant women (highly exposed to phthalate in third trimester of pregnancy) in the U.S., the neurorogical problems in their children had been prolonged even enhanced until 4–9 years old [4]. Another group in risk, is children exposed to phthalates *via* mouthing items as well as breast milk, infant formulas, plastic food container and toys, cups and bowls, and even indoor air. Epidemiological evidence revealed that boys, whose mothers exposed to phthalates during pregnancy, showed an augmented incidence of inborn genital malformations and spermatogenic dysfunction [5]. Regarding to the broad range of phthalate toxicity in human, animals and marines, their distribution in various parts of plants including agricultural and medicinal herbs could be a serious concern.

**Figure 1:**
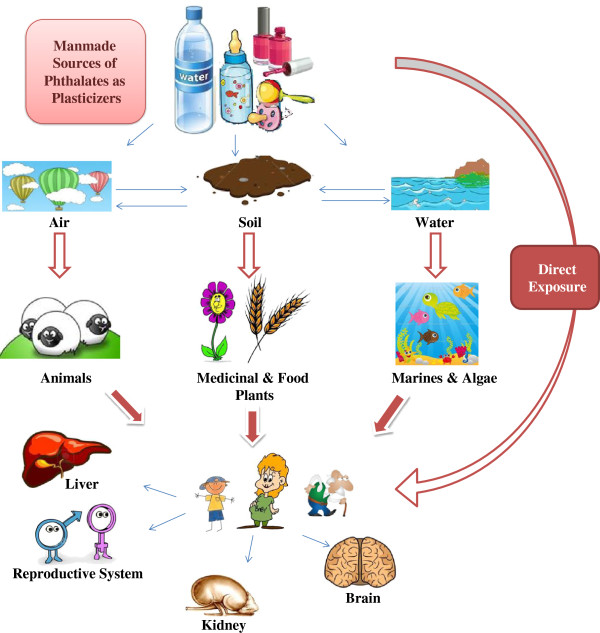
A diagram of the natural circulation, deposition and bioaccumulation of phthalates in relation to human exposure and health effects.

Interestingly, plants receive both nutrients and toxic substances through the roots as well as above-ground green parts. High accumulation of phthalates in stems of some types of crops has been reported [6]. For instance, three important food plants such as agricultural crops (*Triticum aestivum*, *Brassica napus*, *Zea mays*) have been specifically mentioned [2]. Actually in one study on seedlings of radish (*Raphanus salivas*) and wheat (*T*. *aestivum*) exposed to the vapor of DBP, the accumulation of phthalate (106 times per 3 days) was observed significantly in the cuticular and wax layers [7].

Surprisingly, some species of the genus *Phyllanthus*, the famous medicinal plants, have been reported to produce phthalates (bis (2-ethyloctyl) phthalate and bis (2-ethylicosyl) phthalate), which most often exhibited antimicrobial activities [8]. Moreover, phytochemical investigation on flowers of *Calotropis gigantea* led to separation of DEHP. The minimum inhibitory concentration (MIC) of this compound was measured between 13 and 128 μg/mL against *Staphylococcus aureus*, *Bacillus subtilis*, *B*. *megaterium*, *Sarcina lutea*, *Escherichia coli*, *Shigella sonnei*, *S*. *shiga*, *S*. *dysenteriae*, *Aspergillus niger*, *A*. *flavus*, *A*. *fumigatus* and *Fusarium sp*. This compound showed toxicity against *Artemia salina* larvae (IC_50_ = 9.2 μg/mL) too [9]. In addition, the leaves of *Pongamia pinnata*, an Indian medicinal plant, have been reported to consist of bis (2-methylheptyl) phthalate and the mentioned compound exhibited inhibitory activity against White Spot Syndrome Virus (WSSV) [10]. There is an increase in employment of commercial herbal extracts, particularly liquid preparations, which are packaged in plastic containers. Although there are some phyto-analytical techniques for detection and quantification of DEHP in herbal remedies, the quality of these products, regarding to their safety, remains under question [11].

Nevertheless, the presence of phthalates in plant and algae sources might be associated with environmental exposure, production or formation of new brands of phthalates in plants is still in doubt and case of discussion between scientists. Additionally, it is proved that brown algae (like *Sargassum*) can synthesize phthalate esters, but their production process and physiological role have not been clear so far [12]. Dimethyl terephthalate has been also identified as pollutants in various red algae such as *Phyllophora neruosa*, *Acanthophora delilei* and *Hypnea musciformis*, while DBP is isolated from brown and green algae (*Undaria pinnatifida*, *Laminaria japonica*,and *Ulva* sp.) raised a concern that DBP might be generated naturally [13]. The challenge will be raised when many of these plants and marine algae are consumed as food or medicinal resources.

Based on our unpublished data, accumulation of phthalates can occur in some medicinal plants *e*.*g*. *Lythrum*, that are usually grown in water flow in rivers and canals. In such cases, wastewater might be the origin of pollution and phthalate exposure to these plants. Sometimes, high exposure to phthalates resulted in about half part of essential oil extraction, which can cause worries to consume such medicinal plants, crops or vegetables. Although, increasing the capacity for phthalate free plasticizer productions is the first step to restrict the distribution of these toxic manmade compounds, finding the new ways for phthalate absorption from the soil in agricultural fields may have benefits. Also, evaluation and examination of diverse sources of medicinal and food plants to determine the level of phthalate accumulation in their organs are extremely recommended to avoid creating toxicity particularly in reproductive systems.

## Competing interest

The authors declared that there is no conflict of interest.

## Authors’ contributions

Both authors contributed equally to the paper. Both authors read and approved the final manuscript.
